# TAF15 downregulation contributes to the benefits of physical training on dendritic spines and working memory in aged mice

**DOI:** 10.1111/acel.14244

**Published:** 2024-06-14

**Authors:** Yun He, Benju Liu, Fu‐Yuan Yang, Qun Yang, Benke Xu, Lian Liu, Yuncai Chen

**Affiliations:** ^1^ Department of Anatomy, School of Medicine Yangtze University Jingzhou China; ^2^ Health Science Center Yangtze University Jingzhou China; ^3^ Department of Medical Imaging, School of Medicine Yangtze University Jingzhou China; ^4^ Department of Pharmacology, School of Medicine Yangtze University Jingzhou China

**Keywords:** aging, physical running, prefrontal cortex, spine, TAF15, working memory

## Abstract

Moderate physical training has been shown to hinder age‐related memory decline. While the benefits of physical training on hippocampal memory function are well‐documented, little is known about its impact on working memory, which is linked to the prelimbic cortex (PrL), one major subdivision of the prefrontal cortex. Here, we examined the effects of physical training on spatial working memory in a well‐established animal model of physical training, starting at 16 months of age and continuing for 5 months (running wheel 1 h/day and 5 days/week). This training strategy improved spatial working memory in aged mice (22‐month‐old), which was accompanied by an increased spine density and a lower TAF15 expression in the PrL. Specifically, physical training affected both thin and mushroom‐type spines on PrL pyramidal cells, and prevented age‐related loss of spines on selective segments of apical dendritic branches. Correlation analysis revealed that increased TAF15‐expression was detrimental to the dendritic spines. However, physical training downregulated TAF15 expression in the PrL, preserving the dendritic spines on PrL pyramidal cells and improving working memory in trained aged mice. When TAF15 was overexpressed in the PrL via a viral approach, the benefits of physical training on the dendritic spines and working memory were abolished. These data suggest that physical training at a moderate pace might downregulate TAF15 expression in the PrL, which favors the dendritic spines on PrL pyramidal cells, thereby improving spatial working memory.

## Graphical abstract

Prolonged physical running improved spatial working memory in aged mice, which was accompanied by an increased spine density and a lower TAF15 expression in the prelimbic cortex (PrL). Specifically, running affected both thin and mushroom‐type spines on PrL pyramidal cells, and prevented age‐related loss of spines on apical dendrites. Correlation analysis revealed that increased TAF15‐expression was detrimental to spines and working memory. Running downregulated TAF15 expression in the PrL, preserving the spines on PrL pyramidal cells and improving working memory in trained aged mice. However, TAF15 overexpression abolished the benefits of running on spines and working memory.

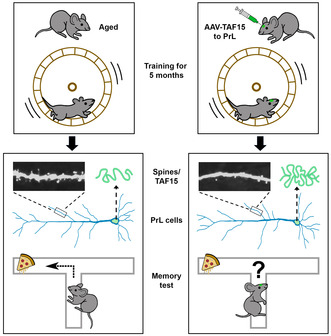

## 1 INTRODUCTION

Age‐related memory decline has a significant effect on the daily lives of elderly people. Whereas safe and effective drug treatment for memory decline that targets specific molecules is challenging (e.g., van Dyck et al., 2023), studies have established the beneficial effects of daily aerobic exercise on memory health. The positive effects of exercise on memory functions have been associated with increased neuronal activation in the prefrontal cortex (PFC) and hippocampus (Damrongthai et al., 2021; Duzel et al., 2016; Jonasson et al., 2017; Stavroulaki et al., 2021; Voss et al., 2013). Running at a moderate pace enhances the PFC activity, which is beneficial to working memory performance (Tsujii et al., 2013). Physical training can also increase the secretion of neurotrophic factors, promote adult neurogenesis, and shift both epigenetic and transcriptomic profiles toward a younger state (Cassilhas et al., 2016; Choi et al., 2018; Fox et al., 2023; Sujkowski et al., 2022; Voisin et al., 2024). Therefore, sufficient training could protect the brain from age‐related changes in neuronal structure, prevent or delay the progression of memory loss, and reduce the risk of various neurodegenerative disorders (e.g., Brown et al., 2019; Kelly et al., 2014; Serra et al., 2022; Sewell et al., 2023; Tsai et al., 2021). Notably, the effects of physical training on memory functions and their cellular basis are primarily derived from the researches on young and adult individuals as well as transgenic animal models (Brown et al., 2019; Cassilhas et al., 2016; Choi et al., 2018; Damrongthai et al., 2021; Kelly et al., 2014; Sujkowski et al., 2022; Tsai et al., 2021; van Praag et al., 1999).

Memory decline in the elderly has been linked to degenerated dendritic structure in the PFC and hippocampus. Studies have reported that cells in the frontal cortex can dynamically modify the synaptic connections between these two brain regions, and the integrity of dendritic spines in the PFC is necessary for memory performance (Ahlbeck et al., 2018; Spellman et al., 2015; Wang et al., 2016). Particularly, the prelimbic cortex (PrL), a major subregion of the PFC in rodents, has been implicated in the processing of spatial working memory (e.g., Moore et al., 2023; Ragozzino et al., 2002; Rossi et al., 2012). Physical training can shape dendritic spines and improve memory performances in the hippocampus. Works from our group have shown that prolonged running at a moderate intensity prevents age‐related spine loss and synaptic collapse in the hippocampus, leading to an improved hippocampal memory function in trained aged animals (Xu et al., 2017, 2019). These benefits have also been reported in a mouse model of Alzheimer's disease (AD) (Xu et al., 2022). While the effects of physical training on hippocampal dendritic structure and memory function have been extensively studied, the impact of physical training on frontal‐associated working memory is less investigated. The molecules that are involved in the structural remodeling of dendritic spines in response to physical training remain largely unclear.

Dendritic spines are extremely plastic in nature and undergo degenerative changes during aging. The stabilization of a dendritic spine largely depends on actin dynamics inside the spine. Studies have reported that a family of proteins known as FET or TET proteins might have a role in regulating actin organization via controlling mRNA transport in spines (Aoki et al., 2012; Fujii & Takumi, 2005), thus can shape dendritic structure and alter synaptic plasticity. The FET proteins were initially discovered as components of fusion oncogenes that cause cancers (Riggi et al., 2007). Among these proteins, TAF15 is a TATA‐binding protein‐associated factor, possessing DNA/RNA binding capacities and participating in transcriptional regulation and microRNA processing (Svetoni et al., 2016). TAF15 may play an important role in spine pathology and memory deficits, as a recent study has identified abnormal aggregates of TAF15 in the brains of individuals with early‐onset dementia (Tetter et al., 2024). In this study, we focused on the pyramidal cells in layers V–VI of the PrL and explored the roles of TAF15 in physical training‐provoked benefits on age‐related spine loss. We found that prolonged physical training initiated at 16 months can improve spatial working memory in aged mice (22‐month‐old), which is correlated with an increased spine density and a downregulated TAF15 expression in the PrL. Overexpression of TAF15 in the PrL via a viral approach abolished the beneficial effect of training on working memory. The preventing effect of training on age‐related spine loss was also eliminated by TAF15 overexpression. These data imply that physical training at moderate intensity may downregulate the expression of TAF15 in the PrL, preventing age‐related loss of spines on PrL pyramidal cells, with an improved consequence for working memory performance.

## 2 MATERIALS AND METHODS

### 2.1 Animals

C57BL/6J wild‐type and B6.Cg‐TgN(Thy1‐YFPH)2Jrs transgenic male mice were used. C57 mice were obtained from the Experimental Animal Center of Hubei Province (Wuhan, China) and Thy1 mice from Jackson Labs (Stock # 003782) (Bar Harbor, ME). These mice were group‐housed in standard cages with ad libitum access to water and rodent chow. All cages were maintained in a temperature‐controlled (22 ± 2°C) and well‐ventilated room on a 12‐h light/dark cycle (lights on at 7:00 AM). All procedures were carried out in accordance with the NIH Guide for the Care and Use of Laboratory Animals. The experiments were approved by the Institutional Animal Care and Use Committees of Yangtze University.

### 2.2 Physical training

Physical training was performed as described previously (Xu et al., 2017, 2022). Mice were initially trained in activity wheels (16 cm in diameter) for 2 weeks (6–10 PM). Mice that had learned to run voluntarily in wheels were randomly assigned to running or control groups. Mice in the running group continued to run for 1 h/day and 5 days a week in the dark. The sedentary controls were placed in immobile running wheels for 1 h/day. Because the running is voluntary, only those with adequate training (Xu et al., 2022) were selected for final data analysis.

### 2.3 Spontaneous alternation task

A T‐maze based task was employed to measure frontal‐dependent spatial working memory (d'Isa et al., 2021; Xu et al., 2023). The maze consists of three arms (35 × 7 cm, 15 cm high) and one central zone (7 × 7 cm), which is illuminated by red light in a dark room. Mice were handled 2 min/day for 5 days to habituate handling prior to the task. The task contains a sample trial (T0) and six test trials (T1‐T6) with a 5 s intertrial interval, and was repeated 4 h later. An overhead camera system (EthoVision, Noldus, the Netherlands) recorded the visits to each arm. For each animal, visiting a goal arm different from one in a previous trial was recorded as a correct alternation, while choosing the same goal arm was scored as an error. The ratio of correct alternations over six trials was calculated as an index of working memory. Choice latency (s) was the time spent entering the goal arm. The maze was cleaned and disinfected with a paper tissue soaked with 30% ethanol.

### 2.4 Rewarded alternation task

A win‐shift spatial memory task was conducted in a T‐maze to measure rewarded alternation ratio (Deacon & Rawlins, 2006; Xu et al., 2023). The maze in the same dimension described above was installed with a food well at the end of each goal arm. Mice were food restricted throughout the experiment to maintain ~90% of their ad libitum body weight. The testing contained a sample trial (T0) and 12 test trials (T1‐T12) and was repeated the next day. In the sample trial, both goal arms were baited with five pellets. During the test trials, rewarded food was provided only in an arm that was not visited in a previous trial. The number of correct entries into baited arms was recorded, and the ratio of correct choices over 12 test trials was used as an index of memory. Latency in picking up the pellets was measured to evaluate animal's decision‐making ability. The time to complete a trial was also measured to assess the locomotor and exploratory activities of animals. The maze was cleaned with 30% ethanol as described above.

### 2.5 Open‐field test

The test was conducted to assess locomotion activity and anxiety‐like behavior (Xu et al., 2023). Mice were handled daily (2 min/day) for 5 days to reduce stress effects during experiments. Testing was carried out in a Plexiglas open‐field arena (45 × 45 cm, with walls of 35 cm height) in a dark room, which is illuminated by red light. The mouse was allowed 10 min of free exploration. Movement was captured by an overhead camera and tracked via ANY‐maze software (v7, Stoelting Co.). The apparatus floor was divided into 16 equal squares (12 outer and 4 inner) for data analysis. The ratio of time spent at the center (four inner squares) was used as an index of motivation.

### 2.6 Golgi staining

Mice were anesthetized with diluted Euthasol containing 50 mg/kg of sodium pentobarbital and perfused via the aorta with 0.9% saline (2 min). The brain tissues were removed and immersed in a freshly prepared impregnation solution (eliteGolgi kit, Bioenno Tech, CA). After 1 day of impregnation in the dark (RT, 22 ± 1°C), the solution was refreshed. Total impregnation time was 5 days. The brain tissues were sectioned coronally at 200 μm using a VT1200 Leica vibratome. The sections were collected in 0.1 M sodium phosphate buffer (PB, pH 7.4) and washed in 0.01 M PB‐saline (PBS) containing 0.3% Triton X‐100 (PBS‐T, pH 7.4), followed by free‐floating staining (5 min) and clarity (2 min) in a parallel manner using the kit. For each brain, sections from the PrL (4–5 sections per brain, Bregma 2.58–1.54 mm) were mounted on gelatin‐coated slides. Sections were dehydrated, cleared, and coverslipped as described (Xu et al., 2022).

### 2.7 Western blotting

The PrL was isolated and collected from prefrontal cortex slices. Dissected tissue was homogenized and processed as described (Xu et al., 2023). Protein samples (20 μg) were separated on 4%–12% Bis‐Tris gel and transferred to nitrocellulose membranes. The membranes were incubated in rabbit anti‐TAF15‐309A (1:20,000; Bethyl), mouse anti‐β actin (1:10,000, AC‐15, abcam) overnight at 4°C, followed by incubation in HRP‐conjugated anti‐rabbit or anti‐mouse IgG (1:10,000; Pierce Biotech) for 1 h at RT. The membranes were developed using SuperSignal (ThermoFisher). The optical densities of protein bands were normalized to respective β actin levels and expressed as the percentages of control group values.

### 2.8 Fluorescent immunostaining (FI)

Mice were anesthetized with a diluted Euthasol as described above and perfused with saline, followed by 4% paraformaldehyde (25 min). The brain tissues were postfixed for 4–6 h at 4°C, cryoprotected in 30% sucrose, and sectioned coronally (20 μm). Unbiased serial sections (1 in 6) were subjected to FI (He et al., 2021; Xu et al., 2018). In brief, free‐floating sections were treated in 0.3% H_2_O_2_ in PBS‐T for 30 min, followed by a blockade of nonspecific sites with 5% normal serum for 1 h. Sections were incubated in mouse anti‐GFP (1:5000; Sigma), rabbit anti‐TAF15 (1:1000; Bethyl, IHC‐00094), rabbit anti‐TAF15‐309A (1:20,000; Bethyl), mouse anti‐NeuN (1:1000; Chemicon, MAB377), mouse anti‐Iba1 (1:2000; Synaptic Systems, #234011), and mouse anti‐GFAP (1:5000; Sigma, cat # 3402) 1–3 days at 4°C. Antibody binding was visualized with anti‐mouse or anti‐rabbit IgG conjugated to Alexa Fluor 543 or 633 (1:200, Molecular Probes). For dual labeling, sections were first incubated in the TAF15 antibody for 3 days at 4°C, followed by incubation in HRP‐conjugated anti‐rabbit IgG (1:1000; PerkinElmer) for 1.5 h and in cyanine 3‐conjugated tyramide (1:150 in 1× amplification; NEL744001KT, Akoya) for 5 min. Sections were rinsed in PBS‐T and then processed for NeuN, Iba1, and GFAP. Antibody binding was visualized with anti‐mouse IgG conjugated to Alexa Fluor 633. Sections were mounted and cover‐slipped with Slide Mount with DAPI (Bioenno Tech, cat# 032125).

The specificity of TAF15 antibodies has been reported previously (Neumann et al., 2011; Tetter et al., 2024) and tested here by pre‐adsorbing the antibodies with a purified recombinant TAF15 protein (5 μg/mL, abcam, ab174418) overnight at 4°C. No staining was observed on the sections when the antibodies were pre‐adsorbed with the recombinant protein (not shown).

### 2.9 Generation of adeno‐associated virus (AAV) and stereotaxic viral injection

AAVs were produced and purified according to published procedures (Guo et al., 2012). In brief, 293 T cells were transfected with pAAV‐TAF15, pHelper, and packaging plasmid (AAV9) at a ratio of 2:1:1. TAF15 coding sequence was subcloned into *Bam*HI/*Hin*dIII restriction sites of pAAV‐CaMKIIα‐EGFP vector (#50469, Addgene). After 72 h transfection, both cells and medium were collected for purification. AAV particles were released from cells with three freeze–thaw cycles, and then treated with 50 U/mL benzonase nuclease (Sigma) and 10 U/mL RNase I (Vazyme) (37°C, 30 min), followed by 0.5% sodium deoxycholate (Sigma) (37°C, 30 min). Cell debris was removed by centrifugation at 2500 × ***g*** for 30 min. Viruses in the supernatant were precipitated by adding 40% PEG8000 and 2.5 M NaCl, and viral pellet was suspended in 0.01 M PBS.

C57 mice were anesthetized with a mixture of ketamine and xylazine (100 and 10 mg/kg body weight, respectively, *i.p*.). AAV‐TAF15 (0.25 μL, titer 1 × 10^13^) or control AAV were delivered bilaterally into the PrL via a glass pipette (tip diameter ~25 μm) and an air pressure injector (Parker's Picospritzer III, Pine Brook). The coordinates used for stereotaxic injections were AP 1.69 mm, ML 0 mm at ±6° oblique, and DV 2.50 mm (Xu et al., 2023). The injection was carried out at a rate of 50 nL/min, and the glass pipette was kept in place for 10 min to prevent virus backflow after the injection.

### 2.10 Dendritic labeling of TAF15‐EGFP‐expressing neurons

The dendritic spines of EGFP‐expressing cells were labeled with DiI (Mol Probes) (He et al., 2021). In brief, 4% PFA‐fixed brain tissues were sliced using a Leica vibratome at RT. Slices (200 μm) were collected and transferred to a holding chamber installed on an upright microscope (Nikon Eclipse) with epifluorescence optics. Dil was loaded in a glass pipette and delivered into designed PrL pyramidal cell (25 μg in 1 μL) with the aid of a three‐axis micromanipulator (Leica) and a Picospritzer (Parker). The pipette was kept in the place for 10–15 min. Slices were kept in 0.1 M PB overnight to allow dye diffusion along neuronal processes.

### 2.11 Image acquisition and spine counting

Three approaches were used to count spines (Xu et al., 2022). (1) The number of spines was counted on reconstructed PrL pyramidal cells in Golgi‐stained sections (three to four cells per brain). Well‐impregnated pyramidal cells were imaged with a Nikon microscope system. High‐magnification (100× oil lens) and z‐stack images (2 μm interval) allowed all spines of a given branch to be imaged. The sides of branches were examined for possible vertical spines. The apical dendrites, including the trunk, obliques in layers II–III, and tuft/distal branches in layer I were analyzed. The basal dendrites residing in layers V–VI were captured and assessed. Spine density was calculated using concentric circles (25 μm) and expressed as the number of spines per 10 μm of segment. (2) Spines in Thy1 brain sections (1 in 6 serial sections) were counted using a stereological fractionator approach. The layers of the PrL were defined under a 5× objective. The stacks of confocal images (z‐stacks) with a step of 1 μm were taken through a 100×/1.4 objective (LSM710), and spines in layers II–III were counted with the aid of Stereo Investigator (MBF Bioscience). Counts of GFP‐labeled dendritic spines (per 250 × 200 × 12 μm) from each section were calculated to obtain a value for each animal. (3) DiI‐labeled EGFP‐positive cells in layers V–VI (3–5 cells per brain and 21–35 cells per group) were selected and imaged (LSM710). Z‐stack images with an interval (1 μm) were reconstructed, and spines on the DiI‐labeled obliques of reconstructed cells were counted. Spines were categorized into thin spines and mushroom‐type spines (Xu et al., 2017, 2018). Stubby spines and filopodia were less observed and were not included in the analysis.

### 2.12 TAF15‐immunoreactive (ir) nuclei

Unbiased serial sections (every 6th) covering the PrL were assessed without knowledge of treatment. The density of TAF‐ir nuclei in PrL layers V–VI was evaluated with a square lattice system with the aid of ImageJ (v2) (Xu et al., 2023). Six sections (20 μm) per mouse were used, and nuclei in an area of 225 × 225 μm^2^ were counted based on stereological principles. TAF15‐ir nuclei were counted only when more than half of the cell nucleus was visualized. The density was expressed as the number of labeled nuclei in a 5 × 10^4^ μm^2^ real area.

### 2.13 Statistical analysis

Data were analyzed using Prism 9 (v9.5.1, GraphPad) and SPSS 22.0 (SPSS Inc.). A one‐sample *t*‐test was employed to distinguish whether explorations of arms were different from those predicted by chance. One‐way ANOVA was employed to detect differences in spontaneous and rewarded alternations and average spine density, followed by Tukey's or Bonferroni's post hoc test. A repeated measures (RM) two‐way ANOVA with trial/day/branch/spine type as a repeated factor was used to compare the choice latency in memory tasks, spine type, and segment‐dependent distribution of spines among the three groups, followed by Sidak's or Bonferroni's post hoc test for pairwise comparisons. Two‐way ANOVA was applied to detect the effects of running on memory performances and spines in AAV‐TAF15 mice, followed by Fisher's post hoc test for multiple comparisons. Pearson's test was used for the correlation analysis, and simple linear regression was conducted with 95% confidence bands shown in graphs. The Kolmogorov–Smirnov test was used to test the normality of data sets, and significance was set at 95% confidence. In bar graphs, data were expressed as mean ± SEM. Box‐and‐whisker plots were also used to represent the distribution of data, with whiskers ranging from the 5th to 95th percentile, while the box depicts the median and the 25th and 75th quartiles.

## 3 RESULTS

### 3.1 Physical training improves frontal‐dependent spatial working memory in aged mice

**FIGURE 1:**
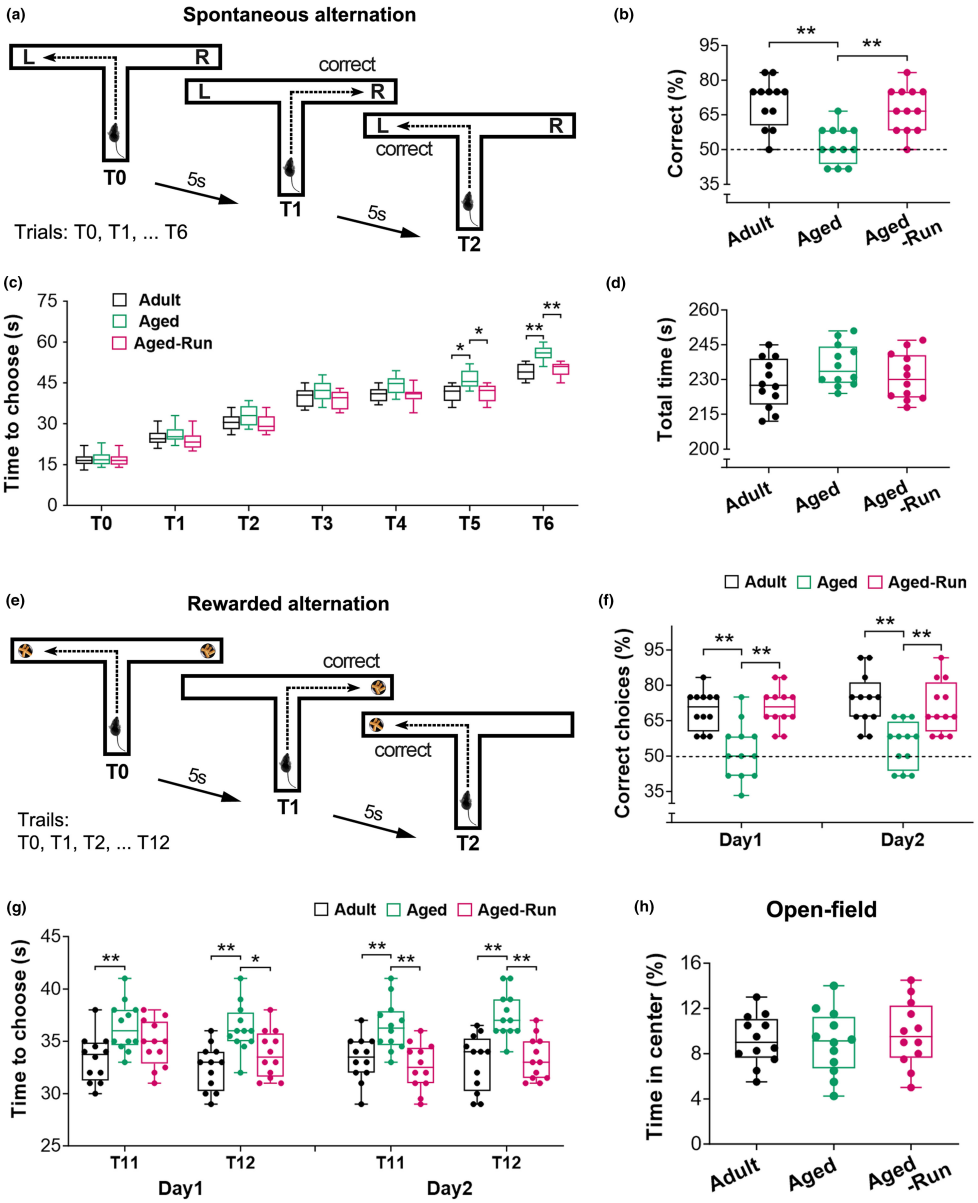
Physical training prevents age‐related decline of spatial working memory. (a–d) Working memory was tested using a T‐maze‐based spontaneous alternation task that contains a sample trial (T0) and six test trials (T1‐T6). A correct alternation was recorded when a mouse visited a goal arm different from its preceding trial, for example mouse visited right arm in T1 compared to the left in T0 (a). The percentage of correct alternations over six trials was used to represent working memory. Both adults and aged runners had better memory performance than aged controls (*F*_2,33_ = 12.81; **p < 0.01; *n* = 12) (b). Choice latency (c) of mice among the three groups displayed an increased trend across the testing trials (*F*_2,33_ = 18.50, *p* < 0.0001). The aged mice exhibited an increased latency at T5 and T6 to make choices compared to the adults (*p < 0.05, **p < 0.01, respectively). However, mice that were trained in running wheels for 5 months (Aged‐Run) demonstrated a lower decision‐making latency versus aged controls (*p < 0.05, **p < 0.01). No difference was found in the total time to complete the trials in the test (*F*_2,33_ = 1.86, *p* = 0.17) (d). (e–g) Improved working memory was evident in a win‐shift rewarded task in the runners. The task contains a sample trial (T0) and 12 test trials (T1‐T12) with an intertrial interval of 5 s (e). In the sample trial, both goal arms were baited with food pellets. During the test trials, rewarded food was provided only in the arm that was not visited in the previous trial. Entries into baited arms were recorded as correct choices. The aged runners displayed a higher percentage of correct choices versus aged controls (Day 1: *F*_2,33_ = 14.17, Day 2: *F*_2,33_ = 10.90; **p < 0.01) (f). The adults also performed better than the aged controls on both trials (post hoc, **p < 0.01) (f). The aged control mice spent a long time to make a choice compared to the runners and adults (Day 1: *F*_2,33_ = 10.44, Day 2: *F*_2,33_ = 13.18; *p < 0.05, **p < 0.01) (g). (h) In an open‐field test, no difference was observed in the time spent at the center among the three groups (*F*_2,33_ = 0.28, *p* = 0.76).

The working memory was assessed via two “stress‐free” T‐maze based tasks (Figure 1). In a spontaneous task (Figure 1a–d), memory decline was apparent in aged control mice (22‐month‐old, *n* = 12). Running for 5 months (Aged‐Run) prevented age‐related memory deficits (*F*_2,33_ = 12.81; **p < 0.01). The aged runners (22‐month‐old, *n* = 12) preferentially visited a new arm rather than returning to a visited arm, showing a higher correct alternation (67.36% ± 2.80) compared to aged controls (52.08% ± 2.32) (Figure 1b). The choice latency of mice in the three groups increased throughout the testing session. At T5 and T6, the aged runners displayed a reduced latency versus aged control mice (*F*_2,33_ = 18.50; *p < 0.05 or **p < 0.01) (Figure 1c). There was no difference in total time to complete the choices among the groups (*F*_2,33_ = 1.86, *p* = 0.17) (Figure 1d), revealing that declined working memory in the aged control mice was not linked to locomotor or exploratory activity.

Improved memory performance in aged runners was further identified in a win‐shift task (Figure 1e–g). They preferred to explore a baited arm, displaying a higher rewarded alternation compared to aged controls at Day 1 and Day 2 (*F*_2,33_ = 14.17 and 10.90, respectively; **p < 0.01). The aged control mice had a lower rewarded alternation compared to adult controls (**p < 0.01), implying age‐related decline in working memory (Figure 1f). Latency to goal arm throughout the testing session was estimated among the three groups (Day1: *F*_2,33_ = 10.44; Day2: *F*_2,33_ = 13.18), with no difference at T1‐T10 (*p* > 0.05), but increased latency at T11 and T12 in aged controls (*p < 0.05 or **p < 0.01) (Figure 1g).

The open‐field test indicated no difference in the motivation of these mice (*F*_2,33_ = 0.28, *p* = 0.76) (Figure 1h).

### 3.2 Physical training prevents age‐related loss of spines on selective dendrites of PrL pyramidal cells

**FIGURE 2:**
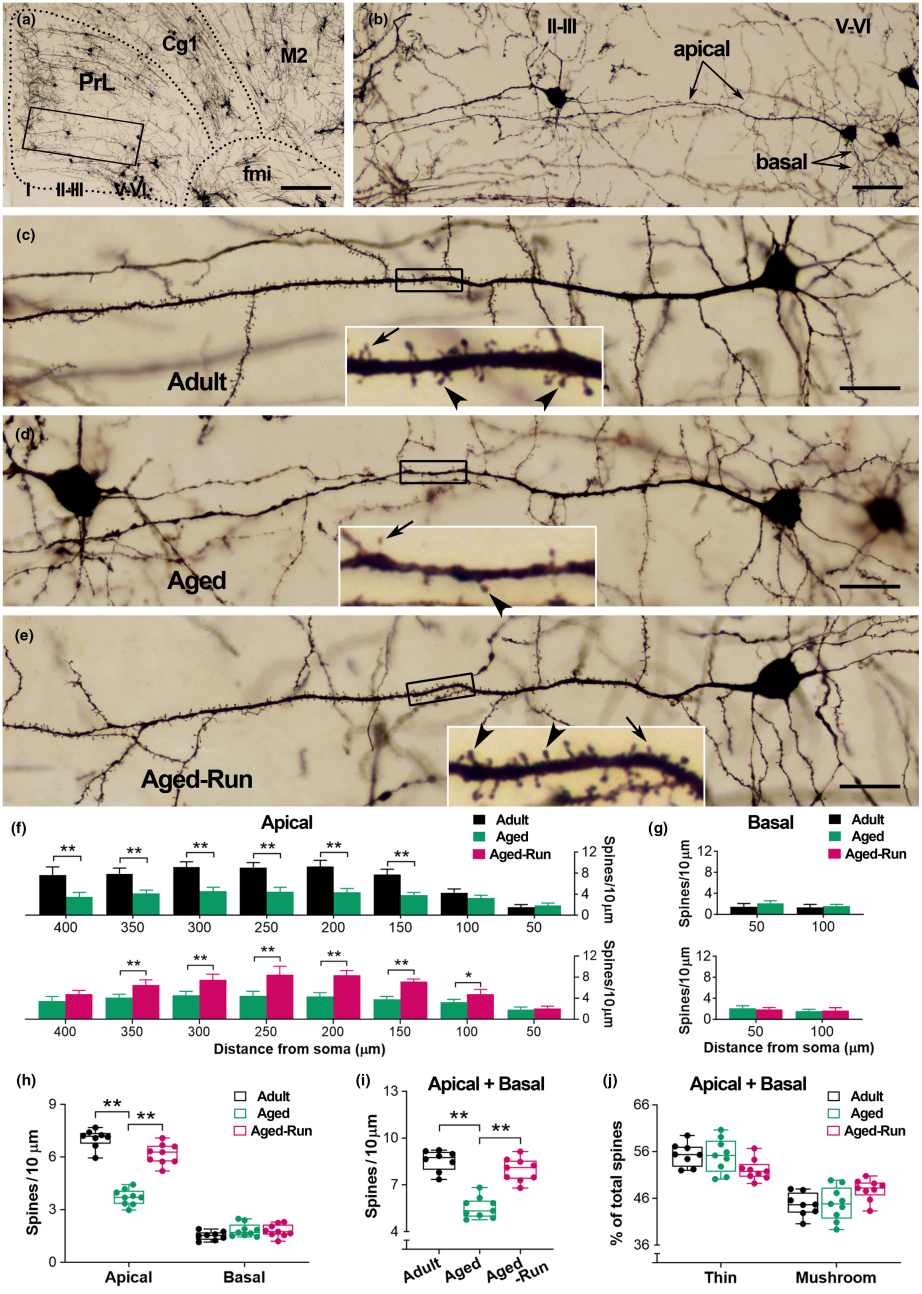
Physical training protects against age‐related loss of spines on PrL pyramidal cells. (a, b) A representative image to show the PrL and adjacent areas in an aged mouse (22‐month‐old). The layers of the PrL are indicated by I, II–III, and V–VI. Boxed area in (a) was magnified in (b) to present the apical and basal dendrites of a PrL pyramidal cell in layers V–VI. Cg1: cingulate cortex; M2: secondary motor cortex; fmi: forceps minor of the corpus callosum. (c–e) Representative PrL pyramidal cells in layers V–VI from an adult, aged control, and aged runner. Boxed segments were amplified to show thin (arrows) and mushroom‐type (arrowheads) spines. Loss of spines on apical dendrites was apparent in the aged control compared to the runner as well as the adult. The PrL pyramidal cell in (d) was derived from image (b). (f, g) On the apical dendrites, age‐related spine loss was observed on segments at 150–400 μm from the soma (Aged vs. Adult), but to a lesser extent in mice that underwent 5 months of physical training (Aged‐Run vs. Adult) (*F*_2,23_ = 94.64; *p < 0.05, **p < 0.01; *n* = 8 or 9) (f). Training prevented spine loss on the apical dendritic segments (Aged‐Run vs. Aged) (*p < 0.05; **p < 0.01) (f). No difference was observed on the basal dendrites (*F*_2,23_ = 1.52; *p* > 0.05) (G). Two‐way RM ANOVA. Error bars in (f) and (g) represent the standard error of the mean (Mean ± SEM). (h–j) Physical training protected against age‐related spine loss on the apical dendrites (*F*_2,23_ = 45.52; **p < 0.01) (h). Aged mice had a lower average spine density compared to the runners and adults (*F*_2,23_ = 45.61; **p < 0.01) (i). The percentage of thin and mushroom‐type spines on both apical and basal dendrites was not affected (j). Scale bars = 250 μm (a), 50 μm (b), and 25 μm (c–e).

To explore the cellular basis of improved working memory in aged runners, the number and type of dendritic spines on the PrL pyramidal cells in layers V–VI were analyzed (Figure 2). Physical training in wheels over 5 months prevented age‐related loss of spines on pyramidal cells in layers V–VI (Figure 2a–e). Because spines on different branches are innervated by different inputs, apical and basal dendrites were separately analyzed (Figure 2f–h). Interestingly, the effect of training on spines was detected on the apical (*F*_2,23_ = 45.52; **p < 0.01; *n* = 8 or 9), but not the basal (*p* > 0.05). On apical dendrites, spine loss in aged sedentary mice was observed on segments at 150–400 μm from the soma (*F*_2,23_ = 94.64; **p < 0.01), the main segments covered with numerous spines. A higher spine density was observed on segments at 100–350 μm from the soma in the runners versus aged controls (*p < 0.05 or **p < 0.01). Average spine density was higher in the aged runners compared to aged controls (*F*_2,23_ = 45.61; **p < 0.01), but comparable with the adults (*p* > 0.05) (Figure 2i). When spines were categorized into thin and mushroom‐type (stubby and filopodia were occasionally observed in the current condition and thus not included), it was found that physical running equally affected these spines, represented by an identical percentage of thin spines and mushroom spines between the runners and aged controls (*p* > 0.05) (Figure 2j). Taken together, physical running protected against age‐related spine loss, benefiting both thin and mushroom‐type spines on the apical dendrites of PrL pyramidal cells.

### 3.3 Working memory correlates with the number of dendritic spines on PrL pyramidal cells

**FIGURE 3:**
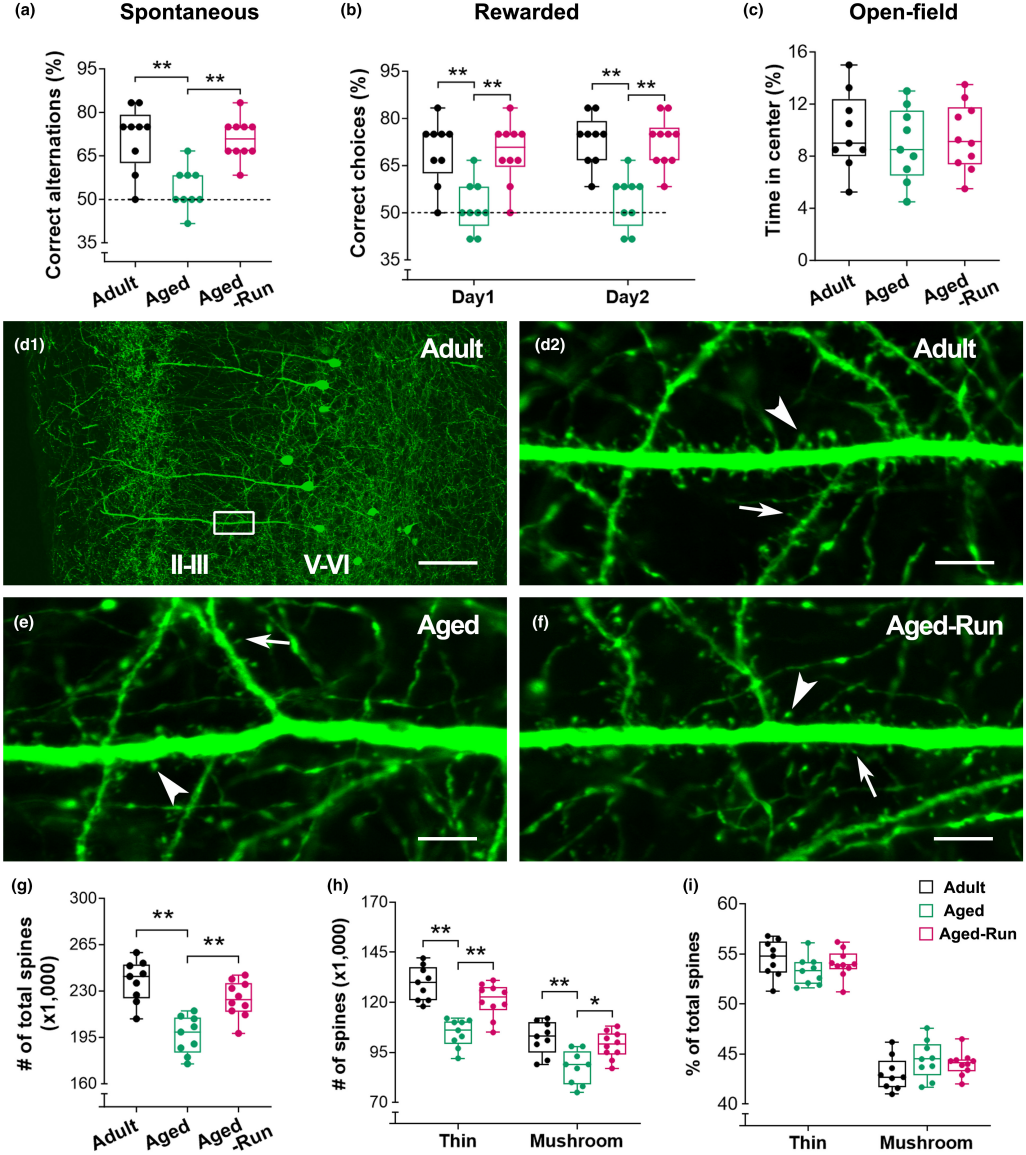
Improved working memory performance and reserved spines on PrL pyramidal cells in a cohort of Thy1‐YFPH aged runners. (a) The mice trained for 5 months (Aged‐Run; *n* = 10) displayed a higher percentage of correct alternations in a spontaneous task versus aged controls (22‐month‐old; *n* = 9), while the later had a lower percentage of correct alternations compared to adult controls (16‐month‐old; *n* = 9) (*F*_*2,25*_ = 12.29; **p < 0.01). (b) Improved memory performance in the aged runners was evident in a 2‐day win‐shift rewarded task. The runners had a higher percentage of correct choices versus aged controls (two‐way RM ANOVA, main effect of running, *F*_2,25_ = 20.16; **p < 0.01). (c) In an open‐field test, no difference was observed in the time spent at the center among the three groups (*F*_2,25_ = 0.29, *p* = 0.75). (d) Representative confocal images from an adult mouse to clarify the layers of the PrL. The cell bodies of Thy1‐expressing pyramidal cells were located in layers V–VI. The dendritic segments covered with numerous spines were mainly observed in layers II–III, where the spines were counted. The boxed area in (d1) was magnified in (d2) to show the dendritic branches with thin spines (arrows) and mushroom‐type spines (arrowheads). (e, f) Representative images from an aged runner and an aged control. Spine loss is apparent in the control. (g–i) Stereological quantitative analysis. Runners had an increased number of spines versus age‐matched controls (*F*_2,25_ = 18.53; **p < 0.01) (g). Training for 5 months protected against age‐related loss of both thin and mushroom‐type spines in the PrL (*F*_2,25_ = 18.02; *p < 0.05, **p < 0.01) (h). No difference was observed in the percentage of either thin or mushroom‐type spines (*F*_2,25_ = 0.99, *p* = 0.39) (i). Counts of GFP‐labeled spines in a volume of 6 × 10^5^ μm^3^. Scale bars = 100 μm (d1) and 7 μm (d2, e, f).

To verify the effect of physical running on spines, a cohort of Thy1‐YFPH mice was used (Figure 3), in which spines on a group of PrL pyramidal cells were YFP‐labeled (Figure 3d–f). The aged runners (*n* = 10) had a higher percentage of correct spontaneous alternations than aged controls (*n* = 9) (*F*_2,25_ = 12.29; **p < 0.01). Improved memory was also observed in the aged runners at a 2‐day win‐shift task (*F*_2,25_ = 20.16; **p < 0.01) (Figure 3a,b). No difference in motivation was detected in an open‐field test (*F*_2,25_ = 0.29, *p* = 0.75) (Figure 3c). These data suggested an improved working memory in 22‐month‐old Thy1‐YFPH runners, which is consistent with the observation in C57 mice (Figure 1).

YFP‐labeled spines were further estimated via unbiased stereological counting (Figure 3g–i). Specifically, spines in layers II–III, where the main dendritic segments of PrL pyramidal cells in layers V–VI are located, were counted. An increased number of total spines was observed in aged runners compared to aged controls (*F*_2,25_ = 18.53; **p < 0.01). Two‐way RM ANOVA suggested that the increased spine number in the aged runners derived from both thin and mushroom‐type spines (*F*_2,25_ = 18.02; *p < 0.05 or **p < 0.01), in line with above Golgi spine data. No difference was observed in the percentage of spine types (*F*_2,25_ = 0.99, *p* = 0.39).

**FIGURE 4:**
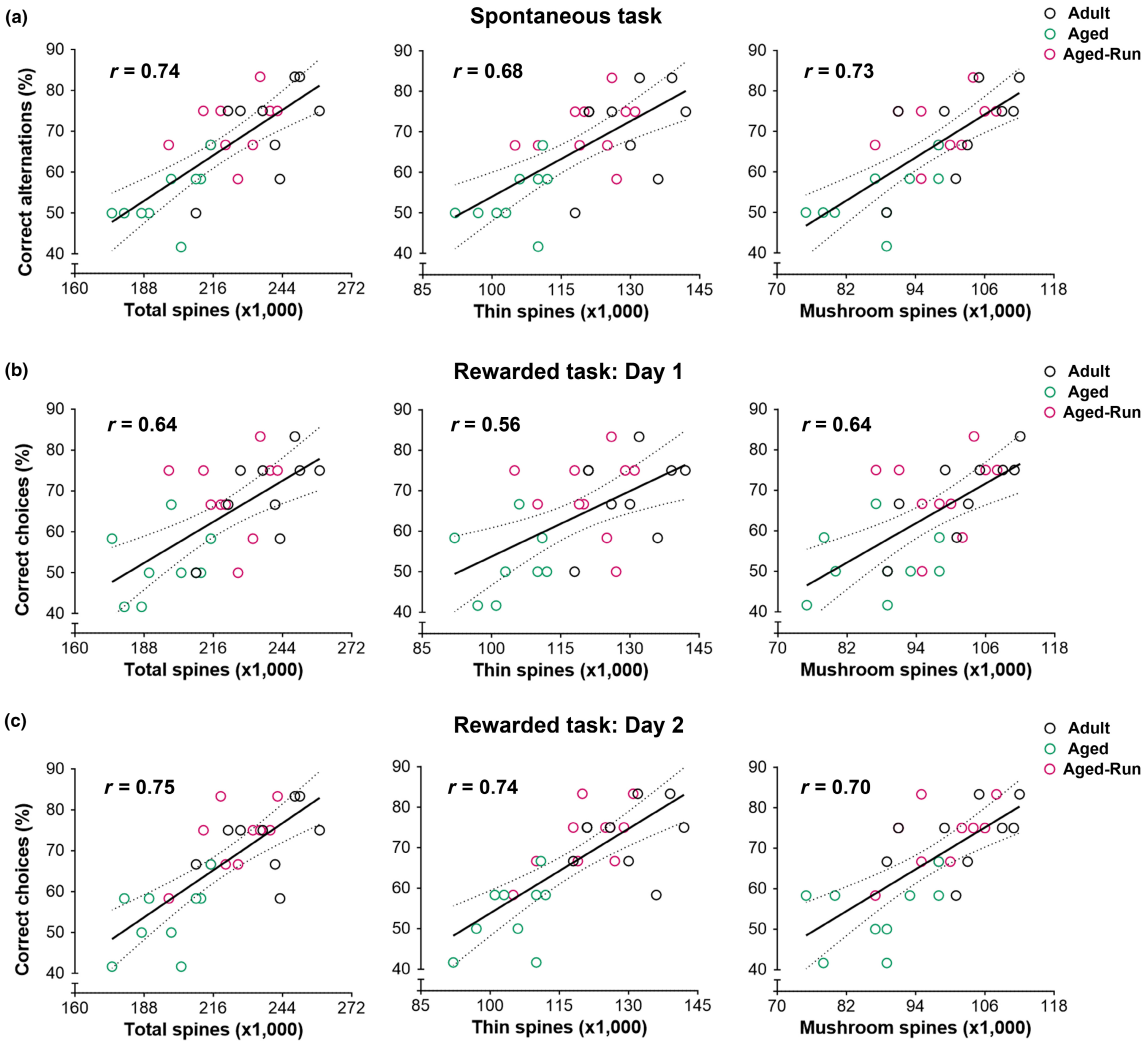
Correlation between YFP‐labeled dendritic spines in the PrL and spatial working memory measured via two T‐maze based tasks. (a) Positive correlations were observed between the correct alternations in a spontaneous alternation task and the numbers of total spines in the PrL (Pearson *r* = 0.74). When dendritic spines were classified as thin or mushroom‐type and analyzed separately, positive correlations were also detected (Pearson *r* = 0.68 and 0.73, respectively). (b, c) In a 2‐day rewarded win‐shift task, the correct choices positively correlated with the number of total, thin, and mushroom‐type spines (Day 1: *r* = 0.64, 0.56 and 0.64, respectively; Day 2: *r* = 0.75, 0.74, and 0.70, respectively; *p* < 0.01), uncovering the critical role of dendritic spines in spatial working memory performance.

The correlation between spines and working memory was analyzed (Figure 4). Spines, including both thin and mushroom‐type, positively correlated with the correct alterations in spontaneous task (Pearson *r* = 0.74, 0.68, and 0.73 for total, thin, and mushroom‐type, respectively; *p* < 0.01) (Figure 4a). The number of spines also positively correlated with the correct choices in rewarded win‐shift task conducted on Day 1 (*r* = 0.64, 0.56, and 0.64 for total, thin, and mushroom‐type, respectively; *p* < 0.01) (Figure 4b) and Day 2 (*r* = 0.75, 0.74, and 0.70 for total, thin, and mushroom‐type, respectively; *p* < 0.01) (Figure 4c). These data indicated that dendritic spines in the PrL are critical for working memory performance in the T‐maze tasks.

### 3.4 Physical training downregulates TAF15 in the PrL, promoting spatial working memory

**FIGURE 5:**
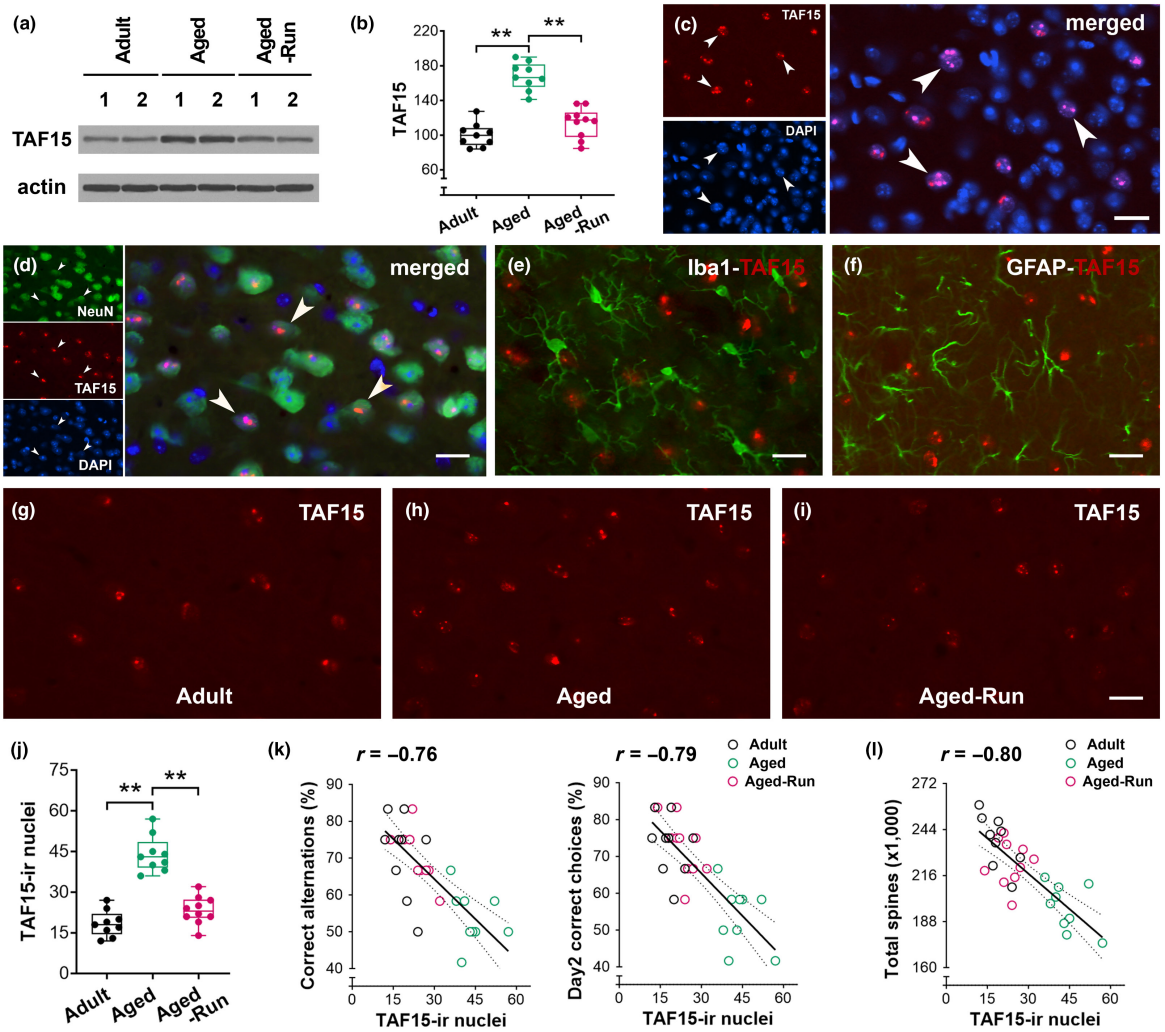
Physical running downregulates TAF15 expression in the PrL of aged mice. (a, b) A decreased TAF15 expression was identified in aged runners (*F*_2,25_ = 46.80; **p < 0.01; *n* = 9 or 10). The protein levels of TAF15 were normalized to respective Actin levels and expressed as a percentage of adult controls. (c) TAF15 was primarily detected in DAPI‐counterstained nuclei. The co‐localization was pointed by arrowheads. (d–f) TAF15 was detected in NeuN‐labeled neurons (arrowheads), but not Iba1‐labeled microglial cells and GFAP‐labeled astrocytes. (g–i) Representative confocal images from an adult, aged control, and aged runner to show TAF15‐ir nuclei in layers V–VI. (j) A decreased number of TAF15‐ir nuclei in the aged runners versus aged controls (*F*_2,25_ = 53.68; **p < 0.01). (k) Negative correlations were observed between the numbers of TAF15‐ir nuclei (per 5 × 10^4^ μm^2^) and the spontaneous alternations (left, *r* = −0.76, *p* < 0.01) as well as rewarded correct choices (right, *r* = −0.79, *p* < 0.01). (l) A negative correlation between TAF15‐ir nuclei and total dendritic spines (*r* = −0.80, *p* < 0.01). Scale bars = 20 μm (c, d–i).

To understand the molecules that may be involved in mediating the effects of physical training on working memory, we measured TAF15 expression in Thy1‐YFPH mice (Figure 5). The protein levels of TAF15 were decreased in aged runners (22‐month‐old, *n* = 10) compared to aged controls (*n* = 9), whereas increased TAF15 expression was apparent in these aged control mice versus the adults (*F*_2,25_ = 46.80; **p < 0.01) (Figure 5a,b). TAF15 was further inspected on immunofluorescence staining sections. TAF15 was mainly detected in the cell nucleus (Figure 5c). Dual labeling with NeuN, Iba1, or GFAP indicated that TAF15 was expressed in neurons rather than in microglial cells and astrocytes (Figure 5d–f). The number of TAF15‐immunoreactive (ir) nuclei was lower in aged runners than aged controls (*F*_2,25_ = 53.68; **p < 0.01) (Figure 5g–j). These data suggested that physical running downregulates the expression of TAF15 in the PrL of aged mice.

The numbers of TAF15‐ir nuclei negatively correlated with correct alternations and choices in the memory testing (*r* = −0.76 and − 0.79, respectively; *p* < 0.01) (Figure 5k), suggesting that a higher TAF15 expression was harmful to memory performance. The correlation between TAF15‐expression and spines in the PrL was further analyzed. TAF15‐ir nuclei negatively correlated with total spines (*r* = −0.80; *p* < 0.01) (Figure 5l) as well as with thin and mushroom‐type spines (*r* = −0.82 and − 0.68, respectively; *p* < 0.01). Taken together, a higher expression of TAF15 in the PrL is detrimental to dendritic spines and working memory of aged control mice. However, physical training downregulates TAF15 expression in the PrL of aged mice.

### 3.5 TAF15 overexpression abolishes the effects of physical training on working memory and dendritic spines

**FIGURE 6:**
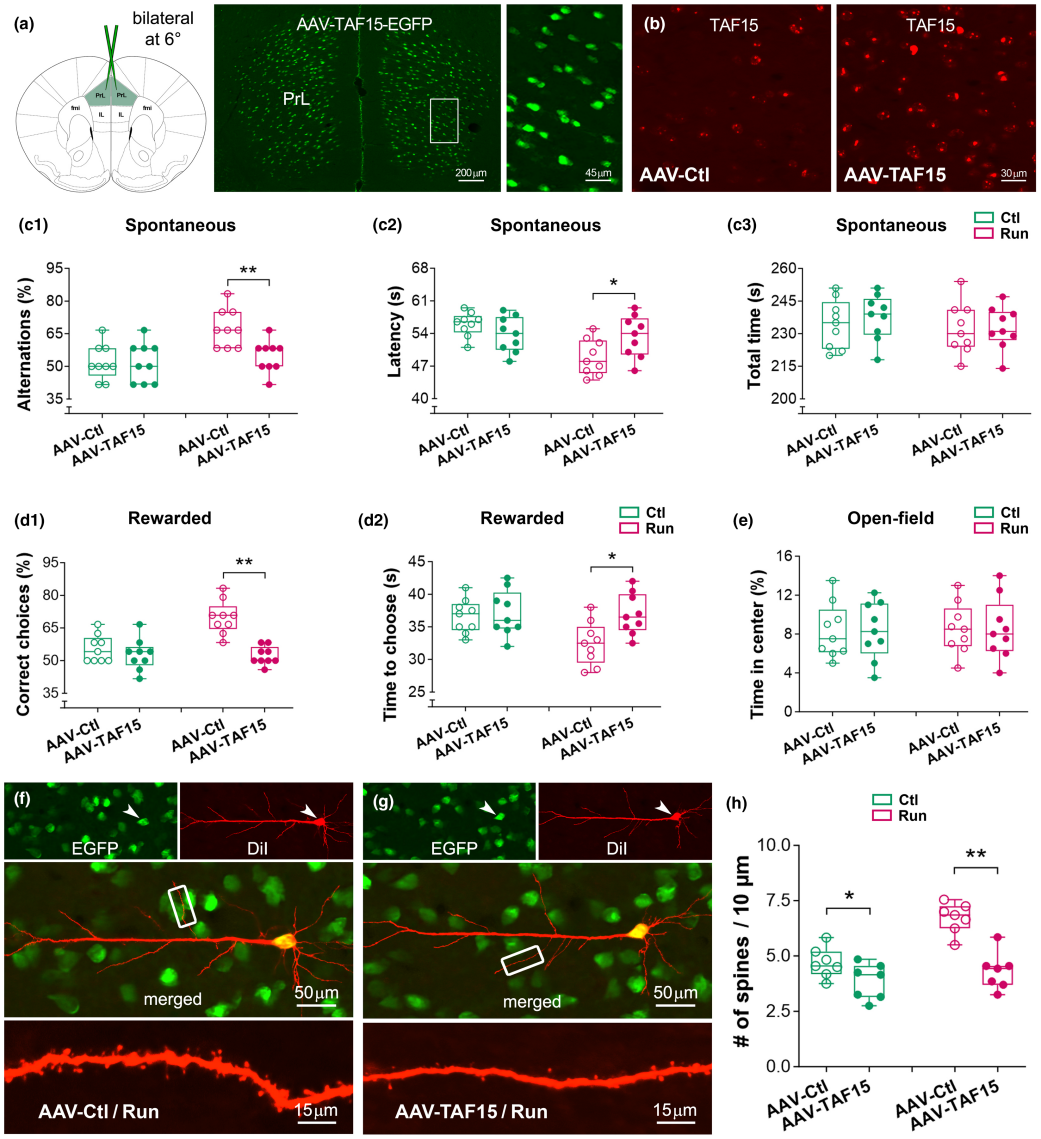
Overexpression of TAF15 abolishes the effects of physical training on working memory and dendritic spines. (a, b) AAV‐TAF15‐EGFP or control AAV‐EGFP was bilaterally injected into the PrL of 16‐month‐old mice, followed by physical training and memory tests, which lasted 20 and 3 weeks, respectively. Robust EGFP‐expressing cells were observed in the PrL. IL, infralimbic cortex; fmi, forceps minor of the corpus callosum. (c) In a spontaneous T‐maze test, runners with AAV‐TAF15 had similar correct alternation (c1) and choice latency (c2) compared to controls (*F*_1,32_ = 11.03, 4.93; Fisher's post hoc, *p* > 0.05; *n* = 9). Improved memory was only observed in runners with control virus (effect of AAV‐TAF15: *F*_1,32_ = 5.40; effect of running: *F*_1,32_ = 11.03; **p < 0.01). No difference in the time to complete the testing (AAV effect: *F*_1,32_ = 0.24; running effect: *F*_1,32_ = 1.03; post hoc *p* > 0.05) (c3). (d) In a rewarded task, the AAV‐TAF15 runners displayed a comparable percentage of correct choices versus their sedentary controls (*F*_1,32_ = 10.28; post hoc, *p* > 0.05) (d1). They also spent equal time to make a choice at the last test trial (T12) (*F*_1,32_ = 4.28; *p* > 0.05) (d2). However, the runners with control AAV had a good memory performance (*p < 0.05; **p < 0.01). (e) No difference in the time spent at the center in an open‐field test. (f–h) TAF15‐EGFP expressing PrL pyramidal cells were labeled with DiI (red) to show their dendritic spines. Representative images were from a runner with control AAV (f) and a runner with AAV‐TAF15 (g). Spine density in the runners with AAV‐TAF15 was lower than those with control AAV (AAV effect: *F*_1,24_ = 32.73; running effect: *F*_1,24_ = 19.22; post hoc, **p < 0.01; *n* = 7). The control mice with AAV‐TAF15 also had a lower spine density versus those with control AAV (*p < 0.05) (h). The benefit of training on spines was only observed in the runners with control AAV (**p < 0.01, not marked in the graph).

To explore the causal link between TAF15 expression and the benefits of physical training on working memory, AAV9‐TAF15‐EGFP or control AAV9‐EGFP was bilaterally injected into the PrL (Figure 6a). Injected mice were grouped into 4 groups (*n* = 9 per group), including aged controls (AAV‐TAF15 vs. AAV‐Ctl) and aged runners (AAV‐TAF15 vs. AAV‐Ctl). The runners were subjected to 5 months of training, followed by memory tests. The efficiency of AAV‐TAF15 expression has been pre‐assessed in an extra cohort of mice (*n* = 3), which shows a ~2.2‐fold increase in the TAF15 protein level (2 months after the viral injection). At the end of memory tests, the levels of TAF15 protein were ~3.6‐fold higher in control mice with AAV‐TAF15 versus AAV‐Ctl (22‐month‐old, *n* = 9). The expression of TAF15‐EGFP was primarily observed in the PrL (Figure 6a,b).

Improved working memory was not observed in the runners with AAV‐TAF15 (Figure 6c1,d1). They had a similar correct ratio compared to the control mice with AAV‐TAF15 in both spontaneous (two‐way ANOVA, main effect of running: *F*_1,32_ = 11.03, *p* = 0.0022; Fisher's post hoc test, *p* > 0.05) and rewarded (running effect: *F*_1,32_ = 10.28, *p* = 0.0030; post hoc, *p* > 0.05) T‐maze tasks. However, the runners with control AAV had an improved memory performance versus the runners with AAV‐TAF15 (Fisher's post hoc test, **p < 0.01). In an open‐field test, we did not find difference in the time spent in the center (running effect: *F*_1,32_ = 0.047, *p* = 0.82; AAV effect: *F*_1,32_ = 0.0002, *p* = 0.98) (Figure 6e), suggesting that overexpression of TAF15 in the PrL specifically affected working memory performance.

To understand whether TAF15 overexpression alters dendritic spines in the PrL, AAV‐infected cells with EGFP‐expression were further labeled with fluorescent dye DiI (Figure 6f,g). Dil‐labeled spines on individual PrL cells were analyzed. Spine data derived from the obliques were used for group comparison, because the above Golgi and GFP data have indicated that the influence of training on age‐related spine changes primarily occurred on the apical dendrites in layers II–III, where obliques represent most of the total length of dendritic branches. The benefits of running on spines were only observed in the runners with control AAV (running effect: *F*_1,24_ = 19.22, *p* = 0.0002; AAV effect: *F*_1,24_ = 32.73, *p* < 0.0001; post hoc, **p < 0.01; *n* = 7). The control mice with AAV‐TAF15 had a lower spine density versus those with control virus (*p < 0.05). The runners with AAV‐TAF15 had a lower spine density, similar to those control mice with AAV‐TAF15 (*p* > 0.05) (Figure 6h). Taken together, TAF15 overexpression in the PrL was able to eliminate the effect of prolonged running on dendritic spines, which may be harmful to working memory performance.

## 4 DISCUSSION

Loss of dendritic spines in the PrL, a subregion of the prefrontal cortex, contributes to a declined working memory in aged animals. We reported here that prolonged physical training via wheel running can prevent age‐related loss of spines, including both thin and mushroom‐type spines on the apical dendrites of PrL pyramidal cells. Importantly, the number of spines positively correlates with spatial working memory performance in two T‐maze based tasks. Furthermore, we found that spine loss and memory deficit in aged mice are accompanied by an increased expression of TAF15 in the PrL of these mice, and physical training is capable to downregulate TAF15 expression, contributing to an improved working memory in aged mice. Finally, overexpression of TAF15 in the PrL abolishes the beneficial effects of physical training on dendritic spines and working memory, pinpointing the detrimental roles of TAF15 overexpression in spine pathology and working memory deficits.

Aging is associated with a variety of changes in memory capacities. Human studies have found that aged adults performed worse than young adults on almost all memory tasks, especially spatial working memory tasks (e.g., Jabès et al., 2021; Pliatsikas et al., 2019; Stavroulaki et al., 2021). Spatial working memory represents the ability to remember recently visited locations. In rodents, spatial working memory is typically detected using maze‐based tasks (Borralleras et al., 2016; d'Isa et al., 2021). Deficits in working memory have been linked to a degenerated neuronal structure in the prefrontal cortex (Benoit et al., 2020; Maharjan et al., 2018; Stavroulaki et al., 2021; Toepper et al., 2014; Vogel et al., 2022). In this study, we found that aged mice (22‐month‐old) had a poor working memory performance in a T‐maze based spontaneous task compared to adult mice (16‐month‐old). The decline of working memory in aged mice was further evident in a food‐rewarded win‐shift task. Interestingly, physical training via wheel running for 5 months can improve the spatial working memory of aged mice in the tasks, which is associated with preserved dendritic spines on pyramidal cells in the PrL.

Declined spatial working memory in aged animals has been associated with age‐related alterations in the properties of pyramidal cells in the prefrontal cortex (e.g., Chang et al., 2022; Moore et al., 2023). The activity of these cells is highly dependent on the integrity of their dendritic spines, the main site of their excitatory synaptic inputs. In the PrL, spines on pyramidal cells in layers II–III and V–VI are targeted by excitatory inputs from different sources, including the adjacent cortex and the hippocampus (Hoover & Vertes, 2007). Here, we focused on the cells in layers V–VI rather than those in layers II–III because they receive monosynaptic glutamatergic inputs from the hippocampus, cooperating spatial memory performance (Kelly & Martina, 2018). Furthermore, these cells are more vulnerable during aging, and age‐related changes on dendritic branches are mainly found in layer V pyramidal cells in the human prefrontal cortex (de Brabander et al., 1998). Studies on rodents have also found age‐related retardation of dendritic branches in the frontal cortex (Dickstein et al., 2013; Sotoudeh et al., 2020). Degeneration of dendrites might result in altered density and/or type of spines. Indeed, loss of spines in the prefrontal cortex is more apparent in aged animals compared to young ones (Young et al., 2014), and the loss of spines occurs in a progressive process, including a 20% loss among middle‐aged rats and a 30% loss among aged rats (Bloss et al., 2011). In the present study, we found a significant loss of spines in aged mice compared to the adults at a middle age. Particularly, the spine loss was mainly observed on the apical dendrites, the possible targets of hippocampal inputs. When spines were categorized into thin and mushroom‐type spines, they were equally affected in the old mice. It is unknown whether external inputs that make synaptic contact with these spines are affected by aging and physical training. Because spines harbor excitatory synapses, increased number of spines in the trained mice might affect synaptic transmission and neuronal circuits, and consequently, improve working memory performance. As reported here, a higher density of spines on the examined pyramidal cells is prominent in layers II–III. The mechanisms responsible for this regional difference require further studies.

Physical training at a moderate pace is beneficial to memory function. Data from human studies support the positive effect of physical training on memory function in elderly people and patients with mild dementia (e.g., Gaitán et al., 2021; Hötting & Röder, 2013; Yu et al., 2021). In rodents, physical training started at an older age can improve spatial learning and memory function (Duzel et al., 2016; Siette et al., 2013; Snigdha et al., 2014). The beneficial effect of training on memory has also been reported in rodent models of neurodegenerative diseases (Choi et al., 2018; Mu et al., 2022; Xu et al., 2022). The underlying mechanisms include enhanced neurogenesis, synaptogenesis, and angiogenesis, as well as the release of neurotrophins in response to physical training (Brockett et al., 2015; Cassilhas et al., 2016; Choi et al., 2018; Sujkowski et al., 2022). In addition, gliogenesis is augmented in the medial PFC after 1 month of physical running in mice (Ehninger & Kempermann, 2003) and rats (Mandyam et al., 2007). Here, we found that declined working memory in aged mice is accompanied by an overexpression of TAF15 in the PrL. AF15 is a 68 kDa TATA‐binding protein‐associated factor, belonging to the TET family of proteins. TAF15 is primarily localized in the nucleus and possesses DNA‐ and RNA‐binding capacities, regulating gene expression both at transcriptional and post‐transcriptional levels (Svetoni et al., 2016). A recent study has reported that TAF15 is involved in the formation of amyloid filaments (Tetter et al., 2024). Importantly, abnormal aggregates of TAF15 in the brain have been linked to the early onset of dementia (Tetter et al., 2024). In the present study, TAF15 immunoreactivity is largely detected in the nuclei of NeuN‐labeled cells rather than in the microglial cells and astrocytes. Increased TAF15 expression is apparent in the PrL of aged sedentary mice compared to the adults. Particularly, the number of TAF15‐ir nuclei in the PrL negatively correlated with the number of spines in this region.

The molecular pathway related to the accumulation of FET protein TAF15 in aged mice remains unclear. Interestingly, physical training downregulates the expression of TAF15 in the PrL, contributing to the preservation of spines on PrL pyramidal cells and enhanced spatial working memory in the trained aged mice. The preservation or stabilization of a spine is dependent on the dynamics of actin filaments inside the spine (e.g., Bonilla‐Quintana et al., 2020). Polymerization of filamentous actin (F‐actin) via actin‐interacting proteins is essential for spine formation and maintenance. In contrast, depolymerization of F‐actin endorses spine retraction and loss. Studies have reported that FET proteins such as fused in sarcoma (FUS) are implicated in the regulation of actin organization via controlling mRNA transport in spines (Aoki et al., 2012; Fujii & Takumi, 2005). FUS‐ and TAF15‐immunoreactive aggregates have been readily observed in the brains of individuals with early‐onset dementia (Tetter et al., 2024). Therefore, increased expression of TAF15 observed in the PrL of aged mice may contribute to the formation of amyloid filaments, which is detrimental to the actin skeleton of spines. Prolonged physical training decreases TAF15 expression in the PrL, leading to a preservation of dendritic spines on PrL pyramidal cells and improved memory function in the trained aged mice. When TAF15 is overexpressed in the PrL via a viral approach, the beneficial effects of physical training are abolished, suggesting that upregulated TAF15 is detrimental to dendritic spines and working memory. TAF15 overexpression also exacerbates age‐related spine loss in sedentary mice, further highlighting the importance of TAF15 in spine pathology. It would be interesting to see if TAF15 knockdown in aged mice could benefit dendritic spines in the PrL, improving spatial working memory. More studies are required to understand the causal link between TAF15 expression and spine loss. TAF15 expression combined with dendritic imaging approach on the same subjects may be able to uncover the role of TAF15 in the collapse of spines.

In summary, physical training at moderate intensity can downregulate the expression of TAF15 in the PrL. Engaging in wheel running over a period of 5 months prevents age‐related retraction of both thin and mushroom‐type spines on selective dendrites of the pyramidal cells in the PrL, which is beneficial to frontal‐dependent spatial working memory. Correlation analysis reveals that TAF15 expression and spine density significantly contribute to the working memory performance in aged mice. These data suggest that physical training can effectively improve spatial working memory in aged individuals, who could benefit from downregulated TAF15 and preserved dendritic spines in the PrL.

## AUTHOR CONTRIBUTIONS

YH, LL, and YCC designed the research; YH, BL, FYY, and QY performed the experiments; YH, BX, and LL analyzed the data; and YH, LL, and YCC wrote and edited the manuscript.

## FUNDING INFORMATION

This work was supported by the Research Joint Development Fund of Yangtze University [WJ2019‐19], Nature Science Foundation of Hubei Province [No. 2023AFB839], and National Natural Science Foundation of China [No. 82271603].

## CONFLICT OF INTEREST STATEMENT

The authors have no conflict of interest to declare.

## Data Availability

Data will be made available on request.
